# If Neuroscience Needs Behavior, What Does Psychology Need?

**DOI:** 10.3389/fpsyg.2018.00433

**Published:** 2018-03-28

**Authors:** Francisco J. Parada, Alejandra Rossi

**Affiliations:** Laboratorio de Neurociencia Cognitiva y Social, Facultad de Psicología, Universidad Diego Portales, Santiago, Chile

**Keywords:** 4E-cognition, complex networks, psychology, neuroscience, mechanistic explanation

Recently, Krakauer et al. (2017) posed an intriguing question about the role of behavior in current neuroscientific endeavors. Ultimately, the authors advocate for a broader conceptualization of neural action when it comes to cracking the code to understand the recursive brain-to-behavior relationship. Even though a skeptic reader might argue that many of the ideas presented and discussed by Krakauer et al. (2017) are not new, it seems relevant to highlight that these are not necessarily sustained and practiced by all brain scientists. It also seems relevant to point at the fact that an important group of researchers might even refuse some of the discussed aspects. Undoubtedly the debate will continue because critical thinking and constant revisiting of procedures and ideas—old and new—is at the heart of any and every healthy scientific effort.

Notwithstanding the potentially deep epistemological void that might separate some scientists when faced to the question posed by Krakauer et al. (2017), Neuroscience keeps striding strong toward identifying systems, levels, components, and describing their organization, relations, and functions using all and every technological, theoretical, methodological, and analytical tool available. Although perspectives, instrumentation, methods and theories might be different, the ultimate search for *mechanisms* unite all of Neurosciences. Let's not forget the infamous theoretical dispute between reticularism and neuronalism—sustained by no others than Camillo Golgi and Santiago Ramón y Cajal—which did not interrupt the advancement and development of modern Neuroscience (Glickstein, 2006). This is something, which does not necessarily happen in Psychology. On the contrary, it seems that in some cases, epistemological differences actually hinder—do no enrich—articulation toward truly interdisciplinary work. Therefore, in the light of the question put forward by Krakauer et al. (2017), here we would like to pose the following question to the reader: *if Neuroscience needs behavior, what does Psychology need?* We will proceed to sketch three pointers that will help us to a possible road toward an answer.

Taking into account the classic emblem “nothing in biology makes sense except in the light of evolution,” we should think of the operation of the brain as inevitably intertwined with—if not determined by—its physical, physiological, psychological, social, cultural, and historical niche. A paradigmatic example of this is the evident relation that circadian rhythms have to solar system dynamics. This is, diurnal mammals such as humans, have been enslaved by the day/night cycle *imposed* to planet Earth from the cosmos. Therefore, it is humbling—to say the least—to think that the omnipresent circadian rhythms are driven by and adjusted to the structure of the wider interstellar context, with deep implications on health, disease, and overall behavior (LeGates et al., 2014); literally a matter of life and death. Thus, *in the light of evolution*, every single physiological and cognitive function could be thought of as the result of each species' phylo- and ontogenetic drift through the ages, time-and-space-locked to the world's regularities. Buzsáki et al. (2013) noted that despite the enormous difference in brain size that can be observed throughout mammalian evolution, the hierarchical organization of brain rhythms is surprisingly well preserved. In other words, regardless brain size, neural interactions unfold over similar time-scales within and across brain networks. Importantly, Buzsáki et al. (2013) suggest that this striking conservation of functional dynamics across mammal species can be explained due to the mechanisms allowing physical movement; the main purpose of the brain/body system. No wonder *minds* have only emerged within systems that can move (Sheets-Johnstone, 2011). At this moment we can draw our first pointer: Psychology needs to abandon purely internalist and/or externalist approaches and re-conceptualize cognition as inevitably *E**mbodied* in biology and *E**mbedded in*, *E**xtended to*, and *E**nacted onto* the world; an approach known as *4E-Cognition* (for a good reference, review the special issue edited by Menary, 2010).

Nowadays, Neuroscience is expanding its focus toward understanding individual and group behavior, exploring all sorts of brain interfaces among other highly interesting topics. However, up until the twentieth century, Neuroscience has successfully focused *exclusively* on studying the components, organization and dynamics within and between systems that are *internal* to the organism. This is, Neurobiology and other Brain Sciences have traditionally study *inward-directed interactions* implemented through complex electro-chemical mechanisms (Kandel et al., 2000; Valenstein, 2006). Chemical dynamics can trigger state changes encompassing milliseconds to several weeks over both short and long spatial scales as well as changes in genetic expression of postsynaptic neurons, signaling—potentially—any kind of change (Brady et al., 2011). Likewise, neuroelectrical dynamics can trigger highly rapid state changes with deep impacts on survival and adaptation (Buzsáki et al., 2013). Combined, electro-chemical activity propagation mechanisms allow the operation of the brain/body system within specific niches. Deregulation or desynchronization of these mechanisms has direct implications on the organisms' functional and structural coupling to the world. Here we can draw our second pointer: Given the inevitably biological embodiment that characterizes cognition, Psychology ineludibly needs to incorporate and integrate insights about *mechanisms enabling inward-directed interactions*, derived from more than two centuries of multidisciplinary Biological and Life sciences.

Despite continuous successful neuroscientific achievements, growing evidence indicates that describing systems parts in isolation and the particular consequences of their selective perturbation is not enough to fully understand behavior in the world (Krakauer et al., 2017). Neuroscience has been a victim of its own success^1^ by reaching a critical point where an enormous amount of information is known about the “processors,” but not so much about “processes” or their relations (Sternberg, 2011; Krakauer et al., 2017). This has happened before: the cell was once thought of as a “bag of enzymes” whose components had to be simply described and mapped in order to be understood. This was a misconception indeed. The cell is now known as a complex dynamic network of interconnected organelles whose changes in state depend on the constant interaction with other cells and cellular environment (Robinson et al., 2007). Just like cell biology once did, Neuroscience has slowly begun moving toward a more integrative approach, considering that an explanation has to be capable of *generating* the phenomenon it tries to explain (Maturana and Varela, 1972, 1984); simply describing or knowing how to perturb the system will not be enough. Neuroscience thus needs behavior in order to understand the operation of the brain as it interacts with the world. Similarly, Psychology has a long history of description and modification of behavior, lacking—however—a model capable of (i) considering neurobiological mechanisms underlying such behaviors and (ii) linking larger regularities, components, and processes articulating interaction with the physical and social niche (including manipulation of novel, known, and meaningful objects, development of family ties, insertion on neighborhood relations, social circles dynamics, etc.). At this stage we can draw our third pointer: Psychology needs to articulate a framework that will allow the discipline to identify and study *mechanisms enabling outward-directed interactions* facilitated by ever-present neurobiological *inward-directed interactions*.

Considering these three pointers, we can start drawing a possible articulation to our original question. Taking into account the complex webs of connections and interactions that range from cellular organelles to neural masses to the whole brain, the inevitable conclusion follows: the nervous system is structurally and functionally intertwined with the living body, while its operation is inexorably restricted and facilitated by the world (Varela et al., 1991; Clark, 1998, 2008, 2013). Thus, the complex *brain/body-in-the-world system* emerges from the dynamic interplay of *inward*- and *outward*-*directed interactions*. In order to understand the topography and function of this complex system, Psychology—as a truly interdisciplinary science—needs to identify its components, connections, and relations; its mechanisms. Psychology needs to grieve and close its historically torturous relation with the traditionally reductionist mechanist explanation and embrace the twenty-first century complex and dynamic mechanicism (Bechtel, 2017); after all, the *nouvelle* cognitive science will need *nouvelle* mechanisms.

We therefore think Psychology needs to empirically and theoretically examine the mechanisms underlying how complex brain/body-in-the-world systems operate both at the topographic and functional levels, from genetic interaction networks to neural networks to socio-cultural networks (Figure 1). This multi-level approach is enabled by ever-advancing data collection techniques that allow exploring the role of ongoing neural activity during perceptual (Zoefel and Vanrullen, 2017), physical (Goldstein et al., 2018), and social (Dikker et al., 2017) interactions. Further enabling the acquisition of large datasets quantifying physiological (e.g., electrocardiography) and behavioral (e.g., motion capture) data (Makeig et al., 2009; Gramann et al., 2014; Parada and Rossi, 2017) within particular social (e.g., socially relations), physical (e.g., geo-referenced location and attributes), and/or virtual (e.g., mobile virtual reality headsets) environments. Thus, exploring high-dimensionality datasets^2^ (Frégnac, 2017), considering an structural approach both theoretical (Bechtel, 2017) and empirically [examining peripheral (Boonstra et al., 2015), central (Shine et al., 2016), and systemic (Kerkman et al., in review) levels], and developing novel conceptual frameworks—like the one proposed by Avena-Koenigsberger et al. (2018) where flexible and time-varying activity propagation in neural systems is facilitated by the dynamic nature of structural interactions—will shorten the gap between biological and social sciences. Importantly, being able to combine these data with phenomenological, etnographical, cross/cultural, cross/species studies will be a major concern as little to no interaction occurs between Psychology and the fields of Anthropology, Compared Cognition, Sociology, etc. Furthermore, visualizing and analyzing high-dimensionality datasets within this context will be extremely challenging. In order to accomplish such a monumental goal, Psychology will have to incorporate Engineering, Art & Design, Informatics and related disciplines within its reach. Thus—in the near future—we envision the implementation of a coherent experimental continuous ranging from highly controlled laboratory experiments (e.g., psychophysics) to semi-structured real-world scenarios (e.g., psychotherapeutic setting) where high-dimensionality data is acquired, critically examined, and modeled as a whole; theoretically using the *4E-cognition* perspective and methodologically relying on Machine Learning, Graph Theory and other modern multivariate analysis techniques.

**Figure 1:**
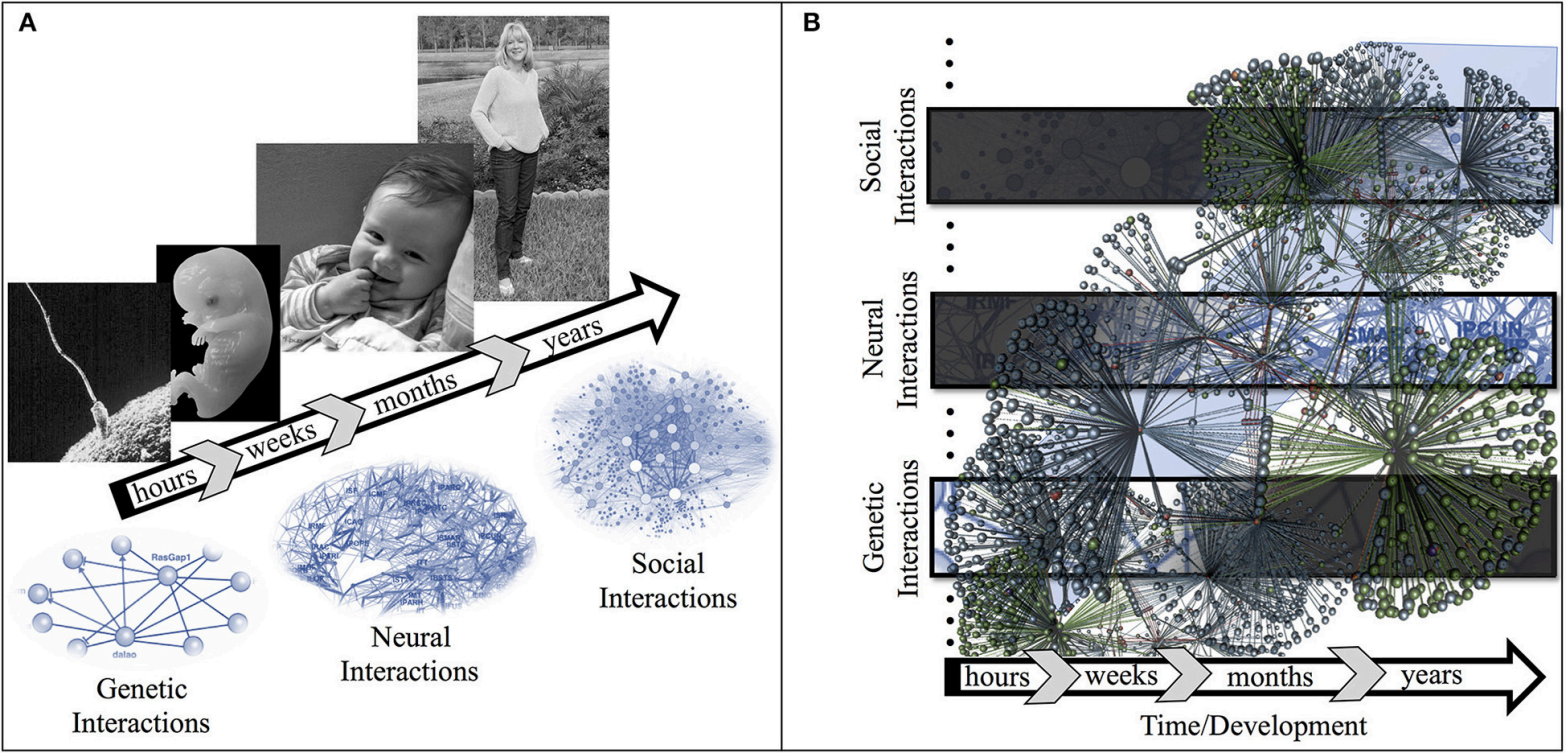
Understanding multi-level structural and functional complex architectures is needed in Psychology. **(A)** Throughout development, different kinds of mechanisms recursively facilitate the gradual increment of complexity in cognitive agents. Relevant deregulation and/or desynchronization within and between intertwined mechanisms will truncate neurotypical development. **(B)** Different levels have diverse roles through development and can unfold their influence at particular time frames, from hours to weeks to years; genetic interactions have more influence early in development, whereas social interactions become ever more relevant as life progresses. It is at the ongoing multi-level interaction where Psychology's main study object, the brain/body-in-the-world system, emerges. We hope novel mechanism can be examined from its conceptualization as a multi-level dynamic mathematical object.

Understanding behaviorally relevant brain function will ultimately depend on our comprehension of large-scale (neuro)physiological dynamics in action within geographical and socio-cultural niches. We suggest reaching this goal will depend on epistemologically and methodologically constructing the brain/body-in-the-world system as a mathematical object—observable and measurable—allowing the integration of the long tradition of network analysis in Sociology and Anthropology with recent advances in Biological and Life sciences; turning Psychology into a *truly* interdisciplinary science. In other words, we think, proper understanding of the complex and dynamical structural and functional scaffold allowing cognition to unfold will be provided by modeling network mechanisms sustaining brain/body systems as they embed, extend, and enact themselves into the world.

## Author contributions

FP conceptualized the present work; FP and AR wrote and edited the present version for publication.

### Conflict of interest statement

The authors declare that the research was conducted in the absence of any commercial or financial relationships that could be construed as a potential conflict of interest.

## Footnotes

^1^Without even mentioning the common and worrying miscommunication of results [de Jong et al. (2016). Responsible reporting: neuroimaging news in the age of responsible research and innovation. *Science and engineering ethics* 22,1107–1130.], the inevitable creation of *neuromyths* [Macdonald et al. (2017). Dispelling the myth: Training in education or neuroscience decreases but does not eliminate beliefs in neuromyths. *Frontiers in psychology* 8, 1314.], and the all-too-often bogus “application” of findings by both *neuro-gurus* and unscrupulous entrepreneurs, practitioners, and researchers [Weisberg (2008). Caveat lector: The presentation of neuroscience information in the popular media. *Scientific Review of Mental Health Practice* 6, 51–56.]

^2^Consider visiting the recently opened Halicioglu Data Science Institute website (http://datascience.ucsd.edu)
